# An Accelerated Concrete Prism Test for Evaluating Fly Ash Mitigation of Alkali–Silica Reaction in Reactive Tuff Aggregates

**DOI:** 10.3390/ma19204335

**Published:** 2026-10-15

**Authors:** Xiaojun Liu, Yan Zhang, Guangye Lv, Wei Li, Xiaojun Huang, Min Deng

**Affiliations:** 1 China General Nuclear Power Corporation, Shenzhen 518000, China; 2 School of Architectural Engineering, Nanjing Institute of Technology, Nanjing 211167, China; 3 College of Materials Science and Engineering, Nanjing Tech University, Nanjing 211816, China

**Keywords:** alkali–silica reaction, reactive tuff, fly ash, concrete prism test, accelerated testing method

## Abstract

The concrete prism test (CPT) retains the aggregate size and specimen configuration of concrete and is widely used to evaluate measures for mitigating alkali–silica reaction (ASR). However, it requires a long test period, and stable control of moist-air curing conditions can be difficult. To improve the efficiency of concrete-scale ASR assessment, four reactive tuffs were investigated using the accelerated mortar bar test (AMBT), standard CPT, and an accelerated concrete prism test (ACPT) involving immersion in 1 mol/L NaOH at 80 °C. Grade I Class F fly ash was used at replacement levels of 0–35%, and the reaction characteristics of the tuff aggregates were examined by microscopy. With 15% fly ash, the 28 d AMBT expansions decreased from 0.212 to 0.376% to 0.036–0.050%, while the 728 d CPT expansions decreased from 0.037 to 0.064% to 0.018–0.029%. All values were below the corresponding expansion limits. The ACPT retained the concrete prism geometry and coarse aggregate grading while accelerating ASR under high-temperature alkaline exposure. The minimum fly ash replacements required to keep all tuff concretes below 0.040% were 20% at 182 d and 30% at 273 d. At 364 d, no replacement level up to 35% met this criterion for all mixtures. As the fly ash replacement increased from 0% to 15% and 35%, the visibly reacted depth of the tuff aggregate decreased from approximately 12 mm to 7 mm and 5 mm, respectively, consistent with the reduction in prism expansion. The results show that ACPT can distinguish the mitigation performance of different fly ash replacement levels within a specified test period. Its expansion criterion should therefore be linked to a fixed assessment age. The 182 d result may be used for rapid dosage selection, whereas the 273 d result provides a stricter recommendation for structures with higher durability and safety requirements.

## 1. Introduction

Concrete structures inevitably suffer from various durability degradation mechanisms during long-term service, mainly including chloride-induced steel corrosion, alkali–silica reaction (ASR), sulfate attack, and freeze–thaw damage [1,2]. Alkali–silica reaction (ASR) is an important expansive durability problem in concrete. Reactive silica in aggregate dissolves in highly alkaline pore solution. The reaction forms products that take up water and expand. This process can crack both the aggregate and the cement paste. It may also reduce stiffness and long-term structural performance [3,4]. For high-reliability and long-lifespan infrastructure such as nuclear power concrete, ASR evaluation must rigorously answer two core engineering questions: whether the adopted aggregate possesses alkali reactivity, and whether the selected mitigation strategy can maintain stable effectiveness under actual material proportions and service environments [5,6,7]. Accurate and reliable ASR assessment methods are therefore essential for durability design of nuclear concrete.

Tuff is widely found in Zhejiang Province, eastern China, and is commonly used as local construction stone. At the nuclear power project studied here, local excavated tuff was considered for concrete aggregate. Its ASR risk and the choice of preventive measures were therefore important engineering issues. Tuff is a pyroclastic rock with a fine and uneven matrix. Its ASR response depends on the type, amount, and location of reactive silica in this matrix. Glassy, cryptocrystalline, and microcrystalline silica may all contribute. Test results can also change with aggregate size, crushing, temperature, moisture, and alkali supply [6,8,9]. Furthermore, aggregate crushing particle size, temperature, humidity, and external alkali supply conditions can significantly affect ASR test results. The tuff samples adopted in this study contain quartz and albite phenocrysts embedded in microcrystalline feldspar matrices, accompanied by a certain amount of microcrystalline quartz. These complex mineralogical characteristics make such tuff aggregates highly representative for investigating the scale dependence and method discrepancy of ASR evaluation.

Supplementary cementitious materials (SCMs), especially Class F fly ash, are the most widely adopted materials for ASR mitigation in engineering practice. The inhibition efficiency of fly ash is comprehensively determined by its replacement dosage, chemical composition, glass phase content, fineness, as well as aggregate reactivity and environmental alkali level [10,11,12,13,14,15,16,17,18]. Different from the simple dilution effect of cement alkalinity, fly ash achieves multi-scale ASR suppression through pozzolanic reactions. It effectively refines concrete pore structure, blocks ion transport channels, reduces pore solution alkalinity, chemically immobilizes free alkali ions, and optimizes the interface reaction characteristics between aggregate and cement paste, thereby inhibiting the generation and expansion of ASR gel [19,20,21,22,23,24,25,26]. Nevertheless, existing studies have shown that highly aggressive accelerated test conditions may lead to inconsistent judgements of effective fly ash dosage compared with long-term field concrete performance. In particular, high-temperature environments and continuous external alkali supplementation in accelerated tests significantly change the reaction kinetics of both aggregates and SCMs, resulting in uncertain applicability of rapid evaluation results in practical engineering.

At present, the accelerated mortar bar test (AMBT) and standard concrete prism test (CPT) constitute the two mainstream standard systems for ASR evaluation. The AMBT features high testing efficiency and strict exposure conditions, adopting crushed aggregates and 80 °C, 1 mol/L NaOH external alkali immersion to rapidly screen aggregate activity and SCM effectiveness. However, aggregate crushing treatment and excessive external alkali supply significantly deviate from actual concrete service conditions, easily causing overestimation of ASR risk and inaccurate judgement of fly ash dosage [5,8,26,27,28,29]. In contrast, the standard 728-day CPT completely retains the original coarse aggregate gradation and concrete mixture proportions, which can truly reflect the ASR expansion characteristics of field concrete and is regarded as the most reliable benchmark method [5,7,27]. Nevertheless, its extremely long test cycle severely restricts the rapid optimization and verification of mitigation schemes in engineering projects. The essential differences in specimen scale, aggregate pretreatment, alkali supply mode, curing temperature, and test duration inevitably lead to inconsistent pass/fail evaluation results between AMBT and CPT, which brings great confusion to the ASR durability design of long-life nuclear concrete structures.

To resolve the evaluation gap between rapid mortar-scale tests and long-term concrete-scale benchmark tests, a series of improved accelerated concrete prism test methods have been developed. Representative methods such as the miniature concrete prism test (MCPT) and RILEM AAR-4.1 adopt elevated-temperature accelerated exposure based on concrete-scale specimens, which can obtain concrete-based evaluation results in a shorter period [30,31,32,33]. Existing studies have verified the feasibility of accelerated concrete tests in evaluating recycled aggregates and composite SCM systems [34,35]. Even so, accelerated results must be checked against long-term or field data [27,36,37,38]. However, current accelerated methods still lack unified evaluation criteria, and the correlation between accelerated results and 728-day standard CPT benchmark data remains unclear, especially for special volcanic tuff aggregates. At present, it is still unknown how evaluation age affects the judgement of effective fly ash dosage, and the applicable scope and limitations of high-temperature accelerated concrete prism tests for tuff aggregates have not been quantitatively clarified, forming an obvious research gap in current ASR evaluation systems.

This study used four reactive tuffs from a nuclear power project in China. Grade I Class F fly ash was used as the preventive material. AMBT, standard CPT, and ACPT in 1 mol/L NaOH at 80 °C were compared. The core objectives of this study are (1) to quantitatively reveal the influence of evaluation age on the effective fly ash replacement level judged by accelerated tests; (2) to compare the consistency of pass/fail ASR evaluation results between accelerated methods and the long-term standard CPT benchmark; (3) to clarify the applicable conditions, advantages, and limitations of the ACPT method for volcanic tuff aggregates. This work aims to provide a scientific reference for the rapid and accurate ASR durability evaluation of tuff concrete in long-life nuclear infrastructure.

## 2. Materials and Methods

### 2.1. Materials

**Figure 1:**
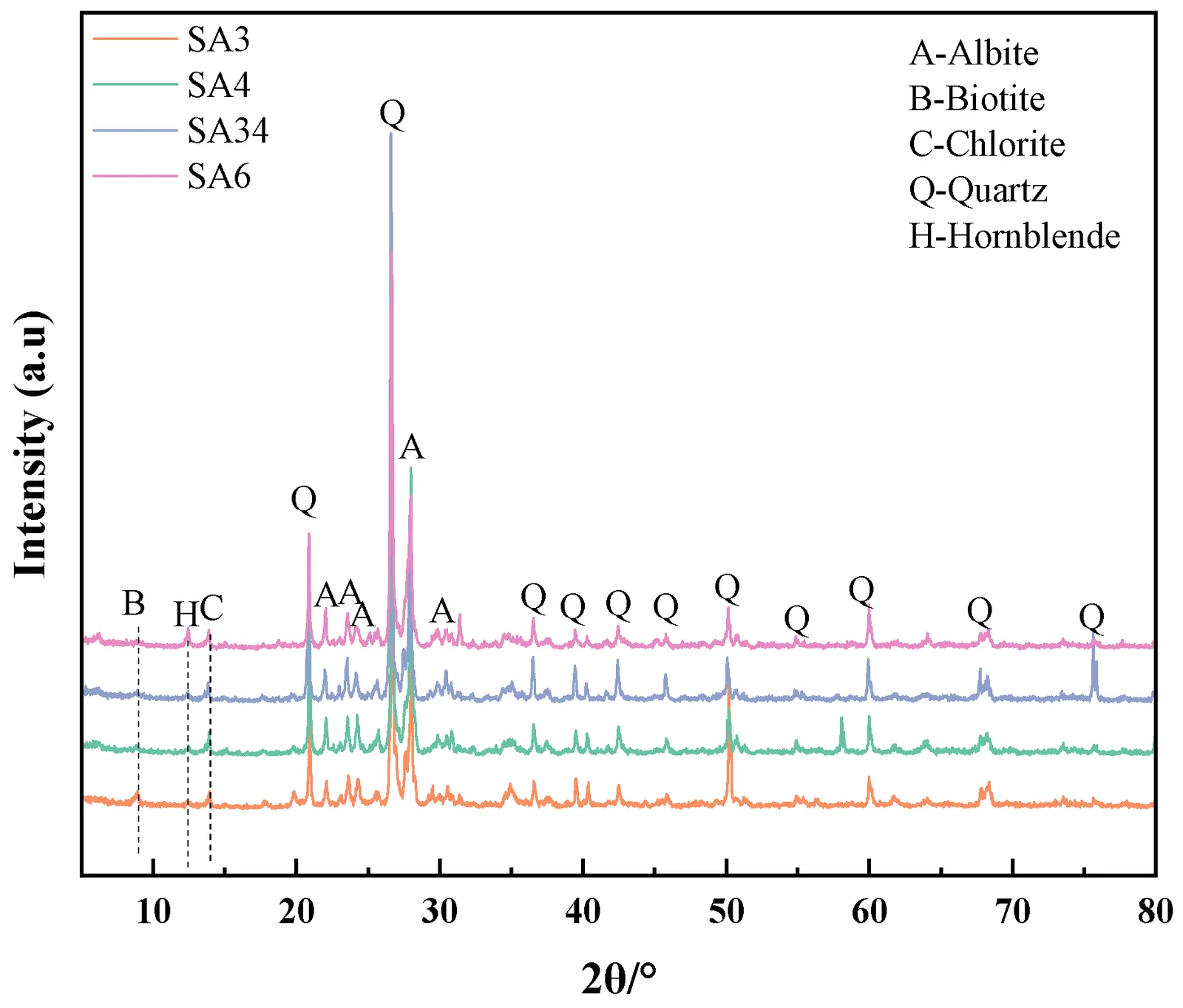
X-ray diffraction patterns of tuff aggregates SA3, SA4, SA34, and SA6.

**Figure 2:**
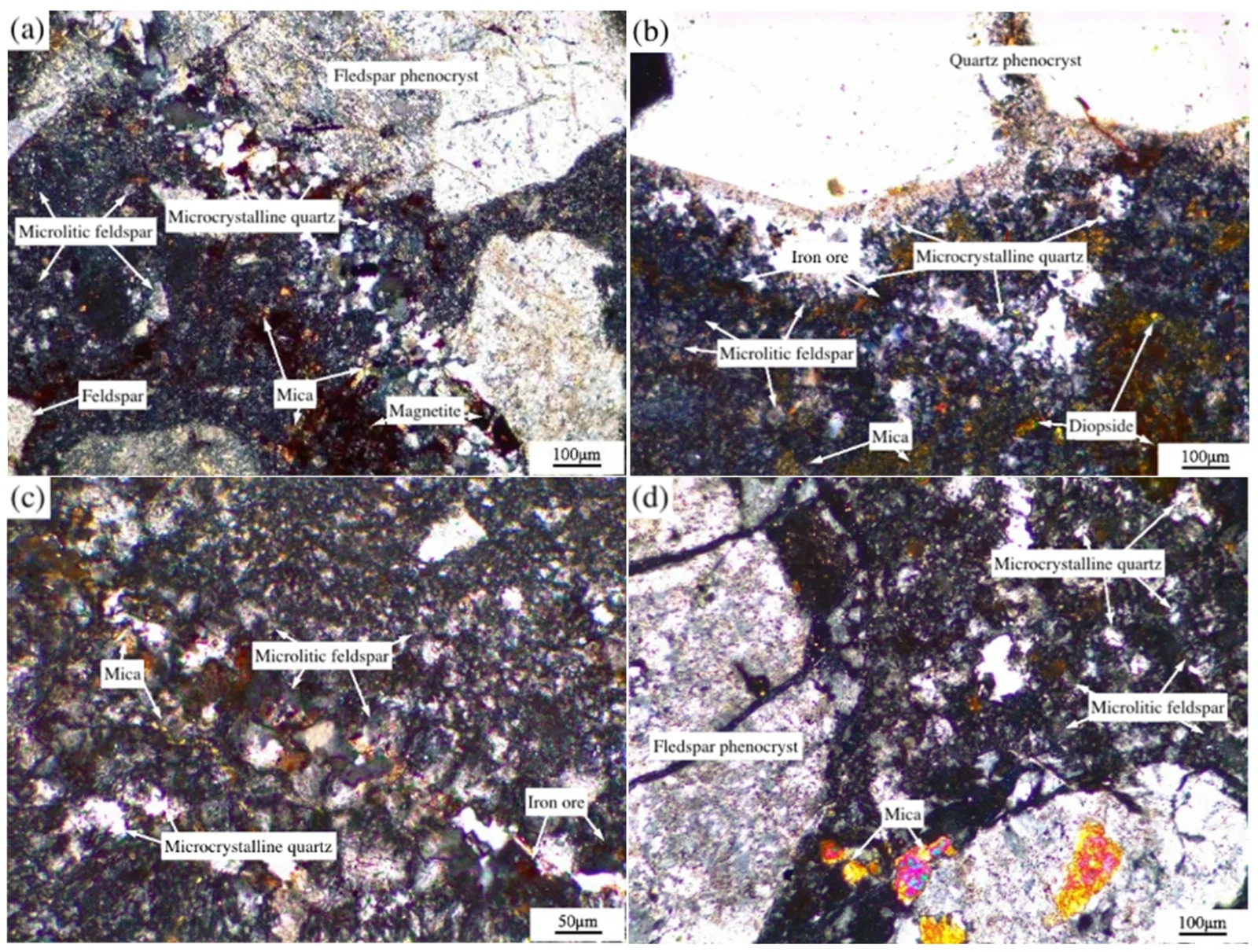
Cross-polarized-light micrographs of tuff aggregates: (**a**) SA3, (**b**) SA4, (**c**) SA34, and (**d**) SA6.

Four tuff aggregates, SA3, SA4, SA34, and SA6, came from different construction zones in Cangnan, Zhejiang Province, China. A nonreactive limestone from Datian, Fujian Province, was used as the control and was named LS. Chemical compositions were measured according to GB/T 176-2017 [39]. The tuffs contained 69.78–71.50 wt.% SiO_2_ and 13.37–15.50 wt.% Al_2_O_3_ (Table 1). Their X-ray diffraction patterns (Figure 1) were dominated by albite and quartz, with minor biotite, chlorite and hornblende. Cross-polarized-light images (Figure 2) showed porphyritic textures containing albite and quartz phenocrysts. The matrix is composed of microcrystalline feldspars, small feldspar crystals, small quartz crystals, muscovite, chlorite, calcite, iron ores and about 3% microcrystalline quartz. The 14 d AMBT expansions of SA3, SA4, SA34, and SA6 were 0.203%, 0.172%, 0.150%, and 0.126%, respectively. LS expanded by only 0.007%. All four tuffs were classified as reactive according to GB/T 14685 [40], while LS served as the non-reactive reference.

**Figure 3:**
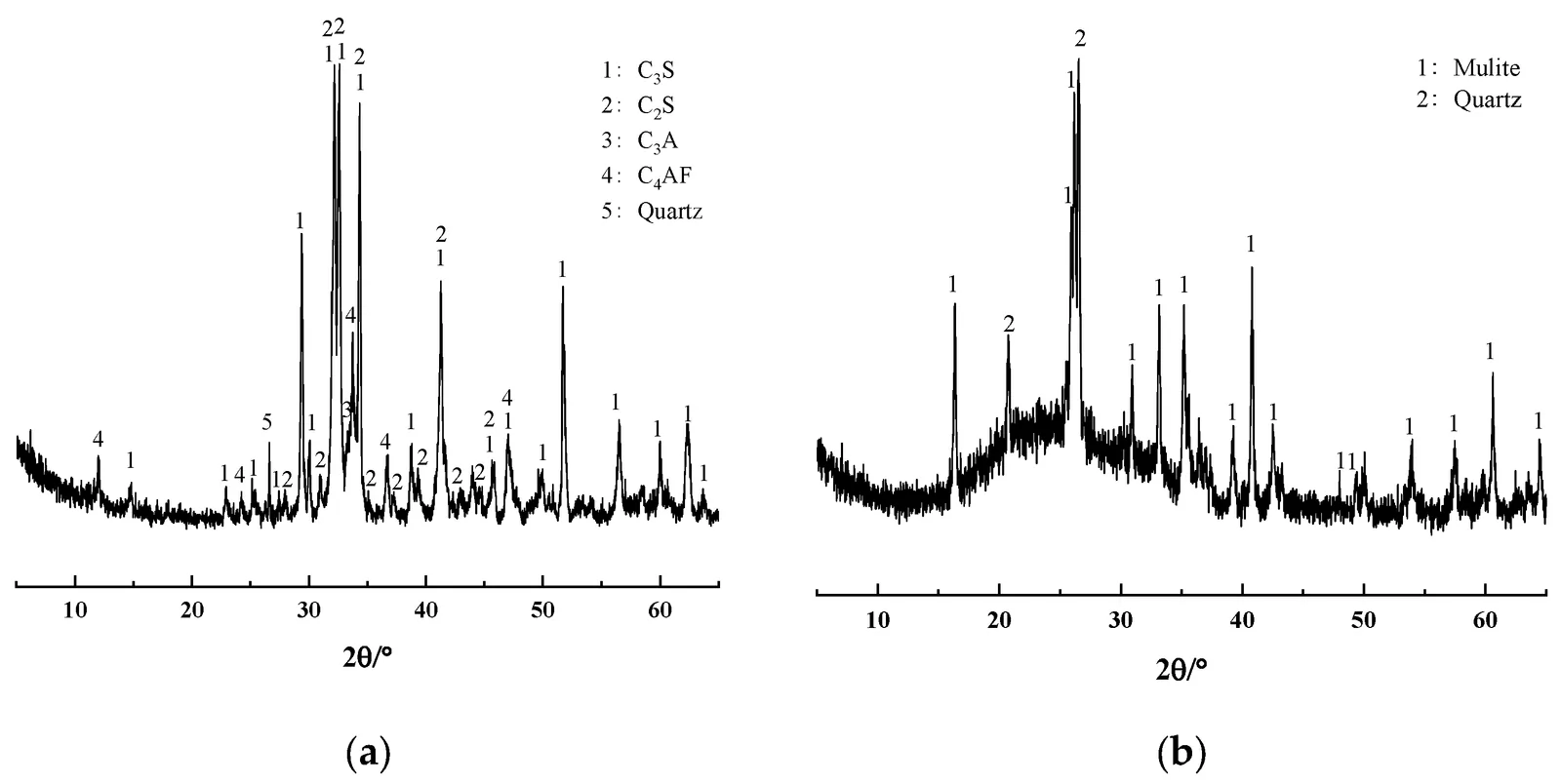
X-ray diffraction patterns of (**a**) cement and (**b**) fly ash.

The cement was a P.N 42.5 low-heat Portland cement from China Resources Cement Holding Limited Company, Zhangping, Fujian, China. The class F fly ash was supplied by Xiamen Yicai Fly Ash Development Company, Xiamen, Fujian, China. The cement contained alite, belite, tricalcium aluminate and ferrite phases, while the fly ash consisted mainly of an amorphous phase with minor quartz and mullite (Figure 3). Their chemical compositions are listed in Table 2. The Na_2_O equivalent alkali contents of the cement and fly ash were 0.38% and 1.43%, respectively.

### 2.2. Test Methods

This study was implemented following a clear sequential research framework. First, raw materials including tuff aggregates, cement, and fly ash were characterized via XRD, XRF, and microscopic observation. Second, mortar and concrete mixtures with different fly ash replacement ratios were designed and prepared. Third, three complementary ASR test methods (AMBT, standard CPT, and ACPT) were performed to continuously measure expansion deformation over designed curing ages. Fourth, statistical analysis was conducted to quantify the influence of fly ash content. Finally, microstructural morphology and elemental composition were analyzed using FESEM-EDS to clarify the inhibition mechanism of fly ash on tuff ASR.

#### 2.2.1. Accelerated Mortar Bar Test

The AMBT followed DL/T 5151 [41], which uses the same 80 °C, 1 mol/L NaOH exposure principle as ASTM C1567 [42]. Each tuff and the control limestone were crushed and separated into the prescribed five aggregate size fractions. Mortar bars measuring 25 mm × 25 mm × 285 mm were prepared with fly ash replacement levels of 0%, 15%, 20%, 25%, 30%, and 35%. After initial curing, the bars were immersed in 1 mol/L NaOH at 80 °C. Expansion was measured at 3, 7, 10, 14, 28, 56, 91, 120, and 150 d. A mixture was judged adequately mitigated when expansion did not exceed 0.10% at the selected age.

#### 2.2.2. Standard and Accelerated Concrete Prism Tests

Concrete prism mixtures followed DL/T 5151, which is similar in principle to ASTM C1293 [43]. The tuffs were processed into 5–20 mm coarse aggregate and sand with a fineness modulus of 2.8. The prisms measured 75 mm × 75 mm × 285 mm. Fly ash replacement levels were the same as those used in AMBT. For the standard CPT, prisms were stored at 38 °C and about 99% relative humidity for 728 d. A second set of prisms was immersed in 1 mol/L NaOH at 80 °C for ACPT. Length change was measured at 28, 56, 91, 126, 182, 273, 364, 455, 546, 637, and 728 d. The limit for both prism tests was 0.040%.

#### 2.2.3. Evaluation Criteria and Comparative Analysis

For each method and age, the minimum effective fly ash level was the lowest tested level at which all four tuff mixtures met the relevant limit. The 728 d standard CPT was used as the concrete-scale reference. For the extended AMBT and ACPT series, the same numerical limits were applied only as comparative screening indices. The minimum fly ash dosage at a given age was the lowest tested replacement for which every reactive-tuff mixture remained below the relevant index. Agreement was calculated for 24 tuff-fly ash combinations: four tuffs and six fly ash levels. It was the percentage of combinations given the same pass/fail result by the accelerated test and the 728 d CPT. A disagreement was called conservative when the accelerated test failed a mixture that passed CPT.

#### 2.2.4. Microstructure Analysis

After curing for designed ages, concrete prisms were sliced and mechanically polished using a Buehler AutoMet 250 Grinder Polisher System (Buehler, Lake Bluff, IL, USA)to produce flat, scratch-free cross-sections. The interfacial microstructural characteristics were preliminarily examined using a Leica polarizing microscope (Leica Microsystems, Wetzlar, Germany). High-resolution microscopic observation and elemental analysis were further performed via a Zeiss Ultra-55 field emission scanning electron microscope (FESEM) (Carl Zeiss Microscopy, Oberkochen, Germany)equipped with an energy-dispersive X-ray spectroscopy (EDS) detector. All specimens were gold-sputtered prior to testing to enhance surface conductivity and imaging clarity. The FESEM tests were carried out in a high-vacuum environment of 1 × 10^−4^ bar with a constant accelerating voltage of 5–10 kV. Observation and EDS testing were primarily focused on the interface between tuff aggregate and cement paste, aiming to characterize ASR gel morphology, microstructural deterioration, and elemental distribution evolution.

## 3. Results and Discussion

### 3.1. Fly Ash Mitigation Evaluated by AMBT

**Figure 4:**
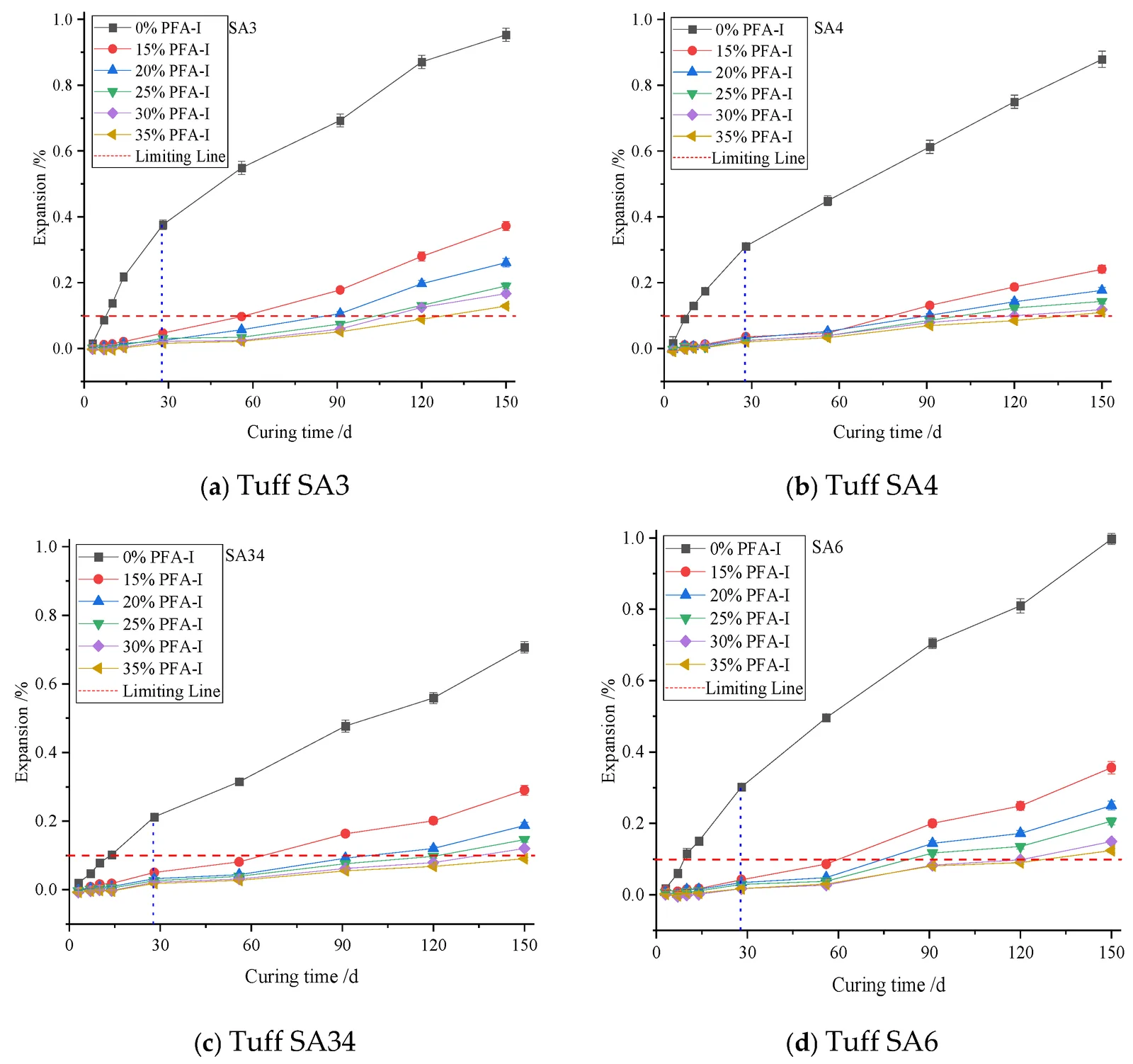
Effect of fly ash replacement on AMBT expansion of tuff aggregates SA3, SA4, SA34, and SA6.

Figure 4 shows the AMBT expansion of the four reactive tuffs and the nonreactive limestone. Expansion increased continuously with exposure time for the reactive aggregates, whereas the limestone specimens remained close to their initial length. Fly ash reduced expansion at every age, and the reduction generally increased with replacement level. Statistical analysis was performed on the expansion data in Figure 4. The mean expansion and standard deviation from replicate specimens were calculated, with standard deviation displayed as error bars.

At 28 d, the control bars made with SA3, SA4, SA34, and SA6 expanded by 0.376%, 0.311%, 0.212%, and 0.302%, respectively. These values were well above the 0.10% criterion, with SA3 showing the greatest expansion. The response confirms the high sensitivity of the AMBT environment to the reactive fine-grained constituents of the tuffs.

At 15% fly ash replacement, the corresponding 28 d expansions decreased to 0.047%, 0.036%, 0.050%, and 0.042%. Thus, 15% fly ash was sufficient for all four tuffs at the conventional 28 d assessment age. Further increases in fly ash replacement produced additional reductions, but the relative benefit became smaller at the higher dosages.

**Figure 5:**
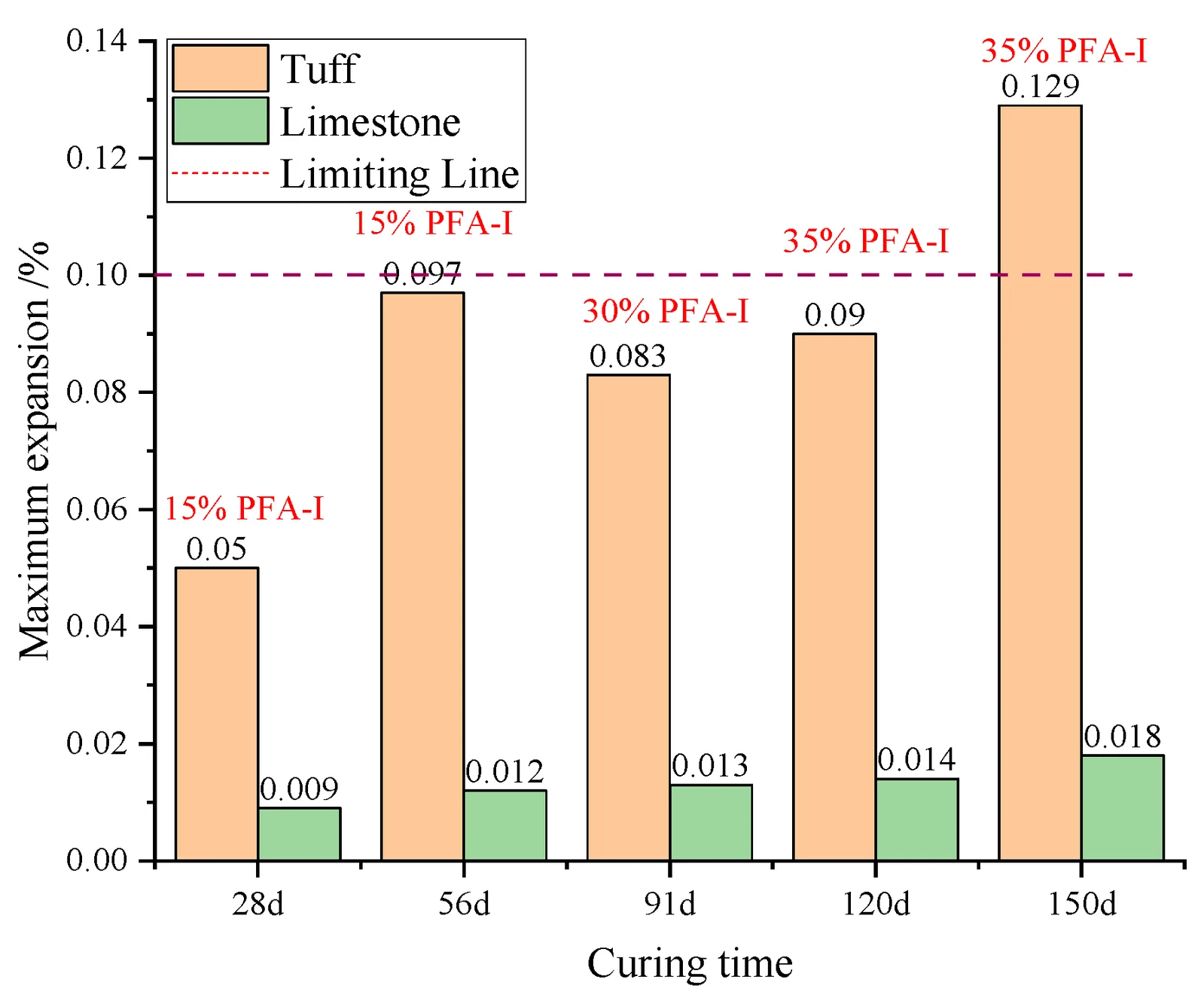
Minimum fly ash replacement required to keep AMBT expansion at or below 0.10% at different assessment ages.

The effective dosage changed when the exposure period was extended (Figure 5). A 15% replacement was still sufficient at 56 d. At least 30% was required at 91 d, and 35% was required at 120 d. At 150 d, even 35% fly ash did not keep all bars below 0.10%. The SA3, SA4, and SA6 mixtures expanded by 0.129%, 0.110%, and 0.124%, respectively.

These results show a clear difference between the usual AMBT age and longer exposure. The 28 d result was a rapid screen and gave the same minimum fly ash level as the 728 d CPT. Longer NaOH immersion increased the apparent fly ash requirement, which increased the safety of the concrete structures.

This mitigation effect arises mainly from the pozzolanic reaction of Class F fly ash, which consumes pore alkali and reduces the alkali concentration available to attack the reactive siliceous phases in tuff. The formation of additional C-S-H gel also refines the pore structure and slows alkali ion transport, consistent with the ASR inhibition mechanism reported in previous studies on siliceous aggregates [21,22]. Nevertheless, the continuous supply of external NaOH in AMBT gradually replenishes the alkali consumed by pozzolanic reactions. This explains why the minimum required fly ash dosage rises significantly with prolonged immersion, an effect that is often overlooked when only the standard 28 d AMBT result is adopted for engineering screening. Most published AMBT studies rely solely on the 28 d expansion criterion; the present finding highlights that extended exposure in the same accelerated environment can overestimate the fly ash dosage requirement for tuff aggregates. This discrepancy is attributed to the fine-grained glassy components unique to tuff, which sustain ongoing silica dissolution under long-term high-alkali immersion.

### 3.2. Fly Ash Mitigation Evaluated by Concrete Prism Tests

#### 3.2.1. Standard CPT

**Figure 6:**
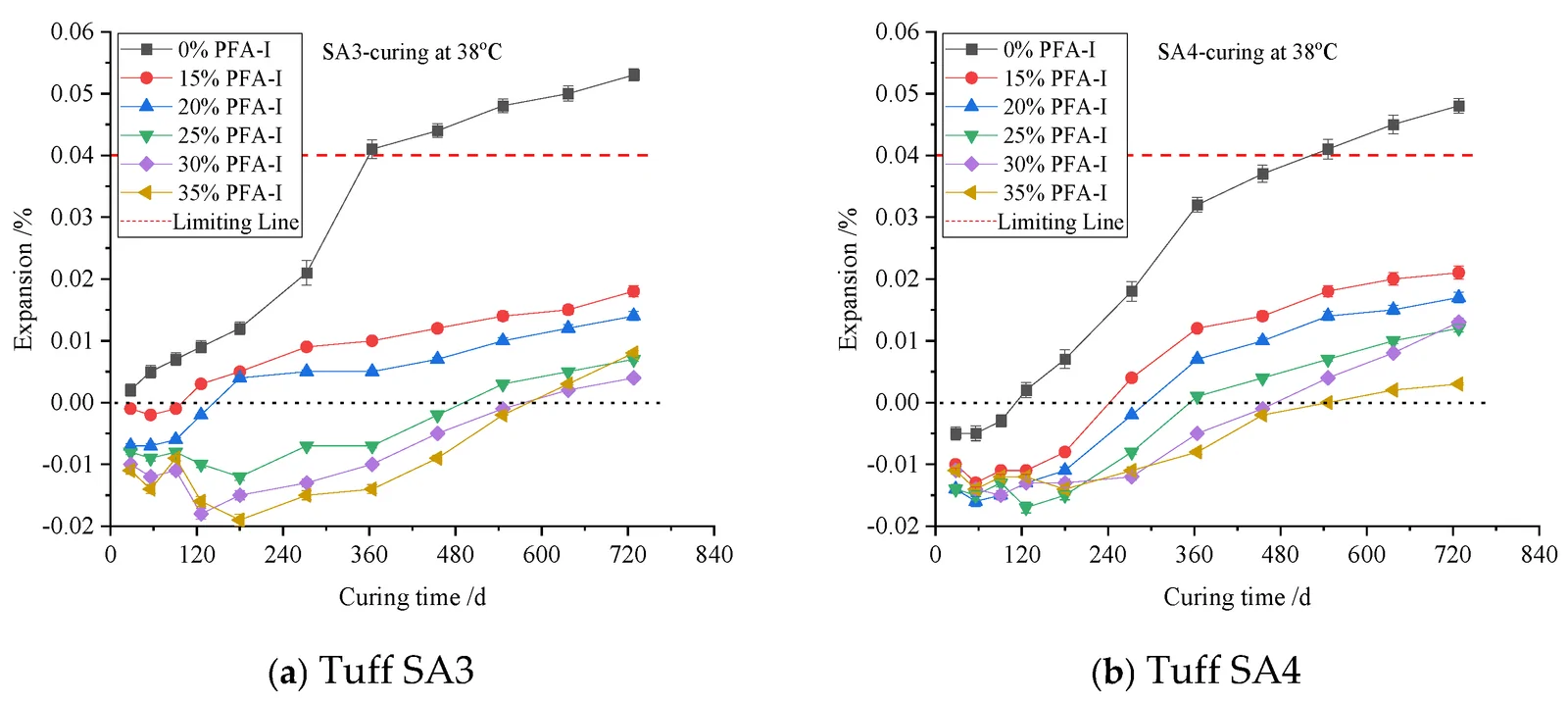

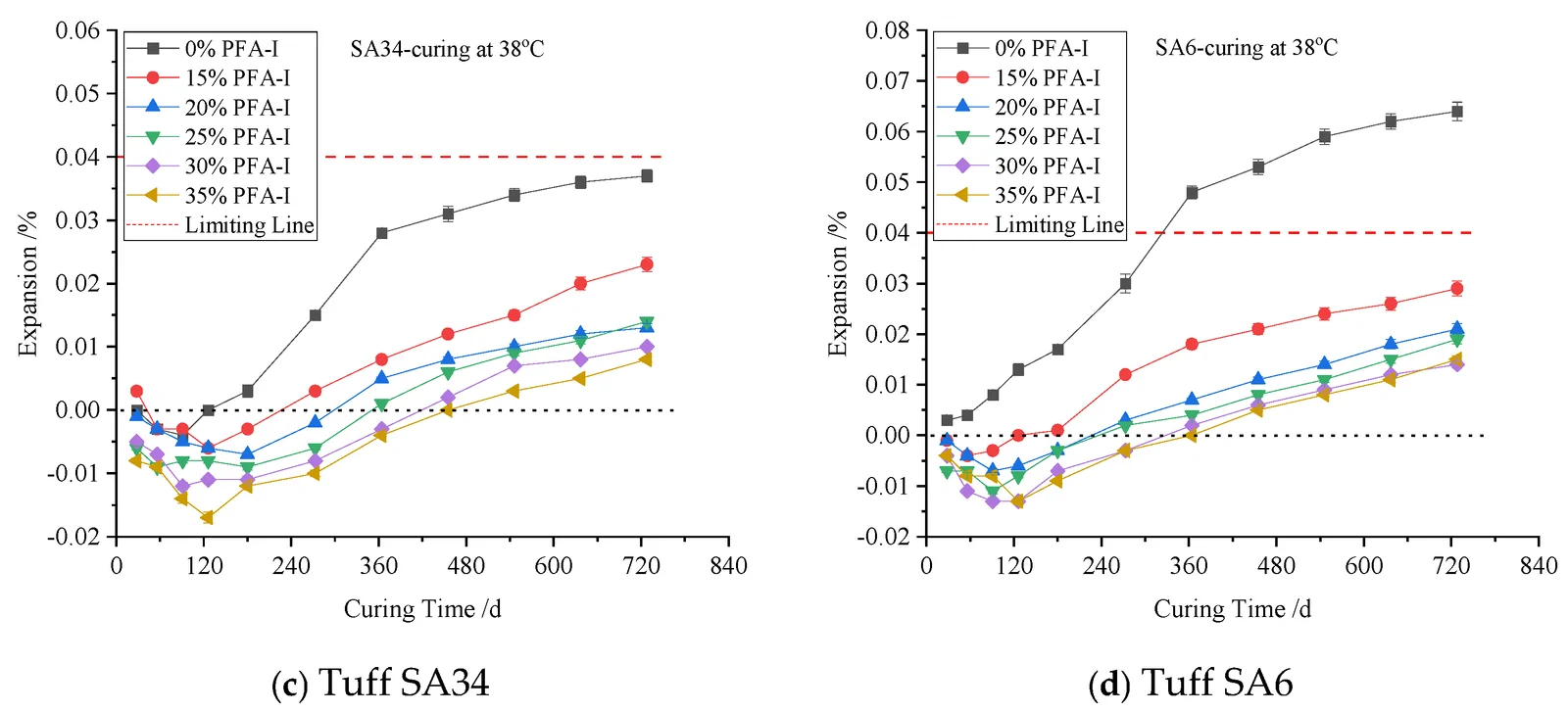
Effect of fly ash replacement on standard CPT expansion of tuff aggregates SA3, SA4, SA34, and SA6 at 38 °C.

Under the standard CPT condition, the concrete prisms initially exhibited slight shrinkage or limited expansion, followed by gradual expansion at later ages (Figure 6). At 728 d, the control prisms with SA3, SA4, SA34, and SA6 expanded by 0.053%, 0.048%, 0.037%, and 0.064%, respectively. Three tuffs exceeded the 0.040% criterion, whereas SA34 approached but did not exceed it.

**Figure 7:**
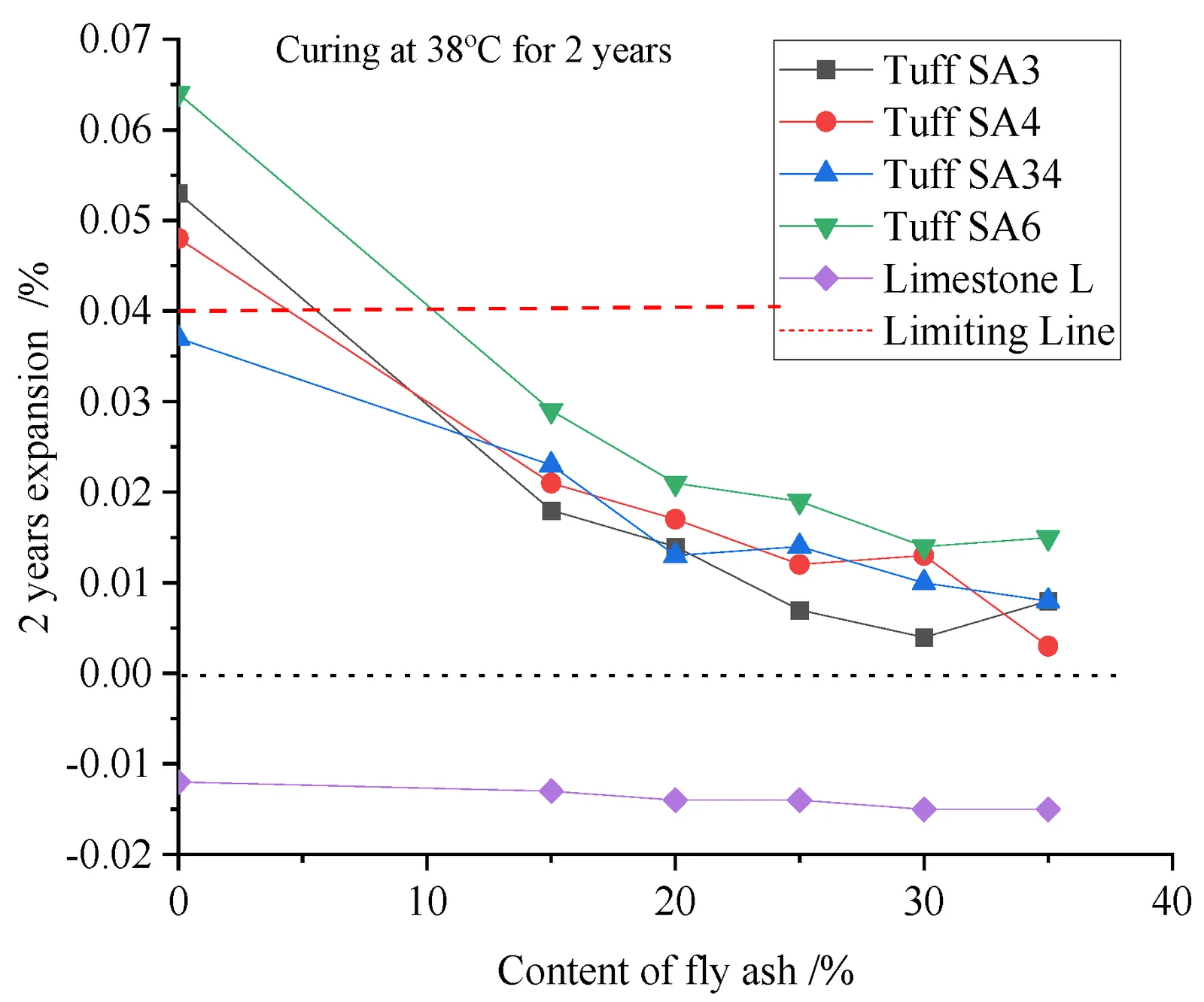
Standard CPT expansion at 728 d as a function of fly ash replacement.

Fly ash reduced the expansion of all tuff concretes. At 728 d, 15% fly ash limited expansion to 0.018%, 0.021%, 0.023%, and 0.029% for SA3, SA4, SA34, and SA6, respectively (Figure 7). All values were below 0.040%. Increasing the replacement to 20–35% generally reduced expansion further, although the incremental benefit was much smaller than the reduction obtained by the first 15% replacement.

The standard CPT therefore identified 15% Grade I Class F fly ash as the lowest tested level that controlled expansion in all four tuffs at 728 d. This result agreed with the 28 d AMBT result. It differed from the results of AMBT at 91–150 d.

Compared with AMBT, the standard CPT uses full concrete prisms with retained coarse aggregate grading and no external alkali supply, which better mimics the internal alkali balance of field concrete. The fly ash pozzolanic reaction continuously consumes alkalis released by cement hydration, permanently lowering the pore solution alkalinity without continuous alkali replenishment. This explains why 15% fly ash remained effective over the full 728 d test period in standard CPT. This observation is consistent with the long-term ASR mitigation trends for volcanic aggregates reported by [11]. The diminishing marginal benefit beyond 15% replacement indicates that once pore alkali is sufficiently depleted, further fly ash addition provides only minor additional inhibition. Such a threshold effect has rarely been quantified for tuff aggregates, which is a distinctive finding of this study.

#### 3.2.2. Accelerated Concrete Prism Test

**Figure 8:**
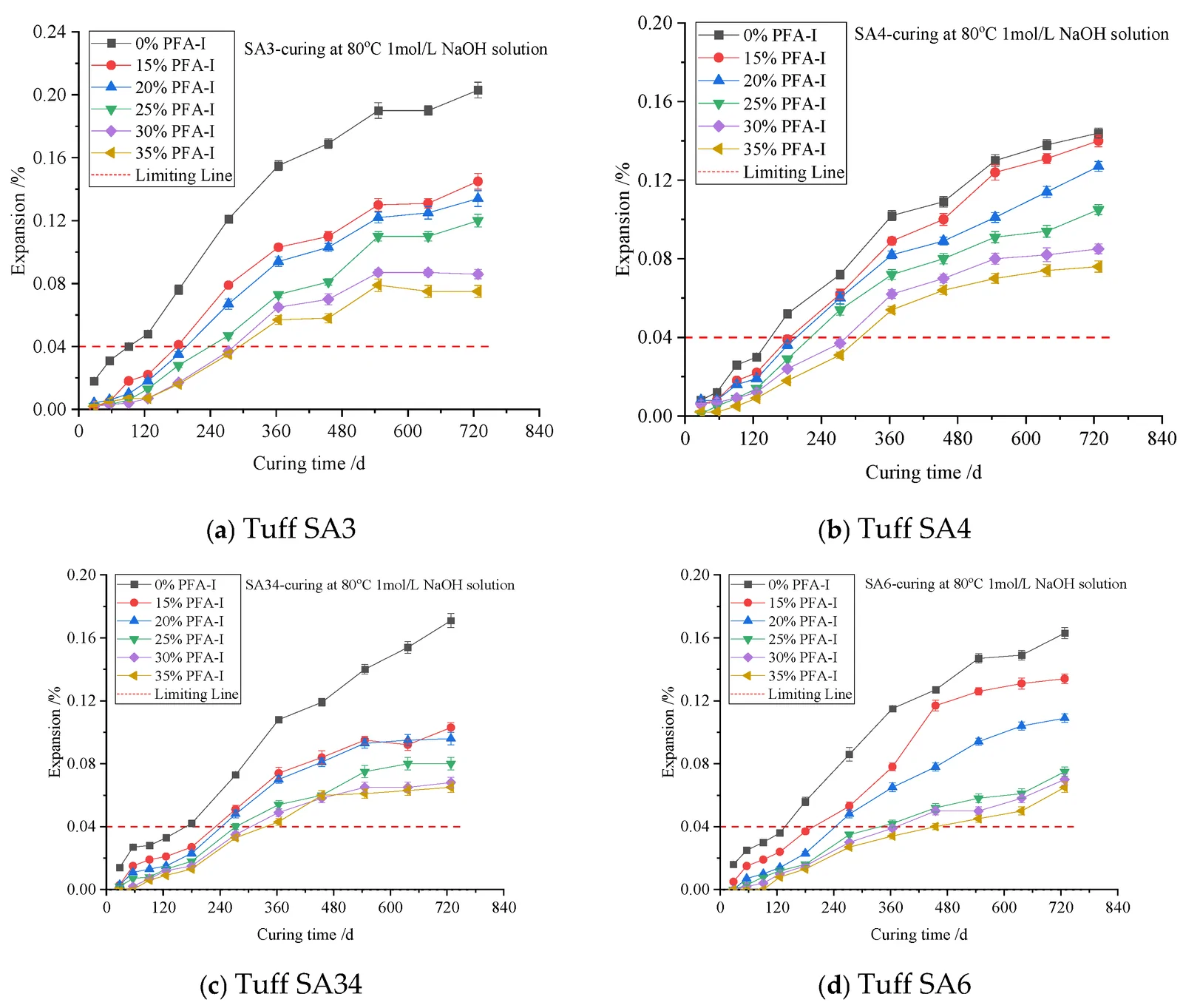
Effect of fly ash replacement on ACPT expansion of tuff aggregates during immersion in 1 mol/L NaOH at 80 °C.

**Figure 9:**
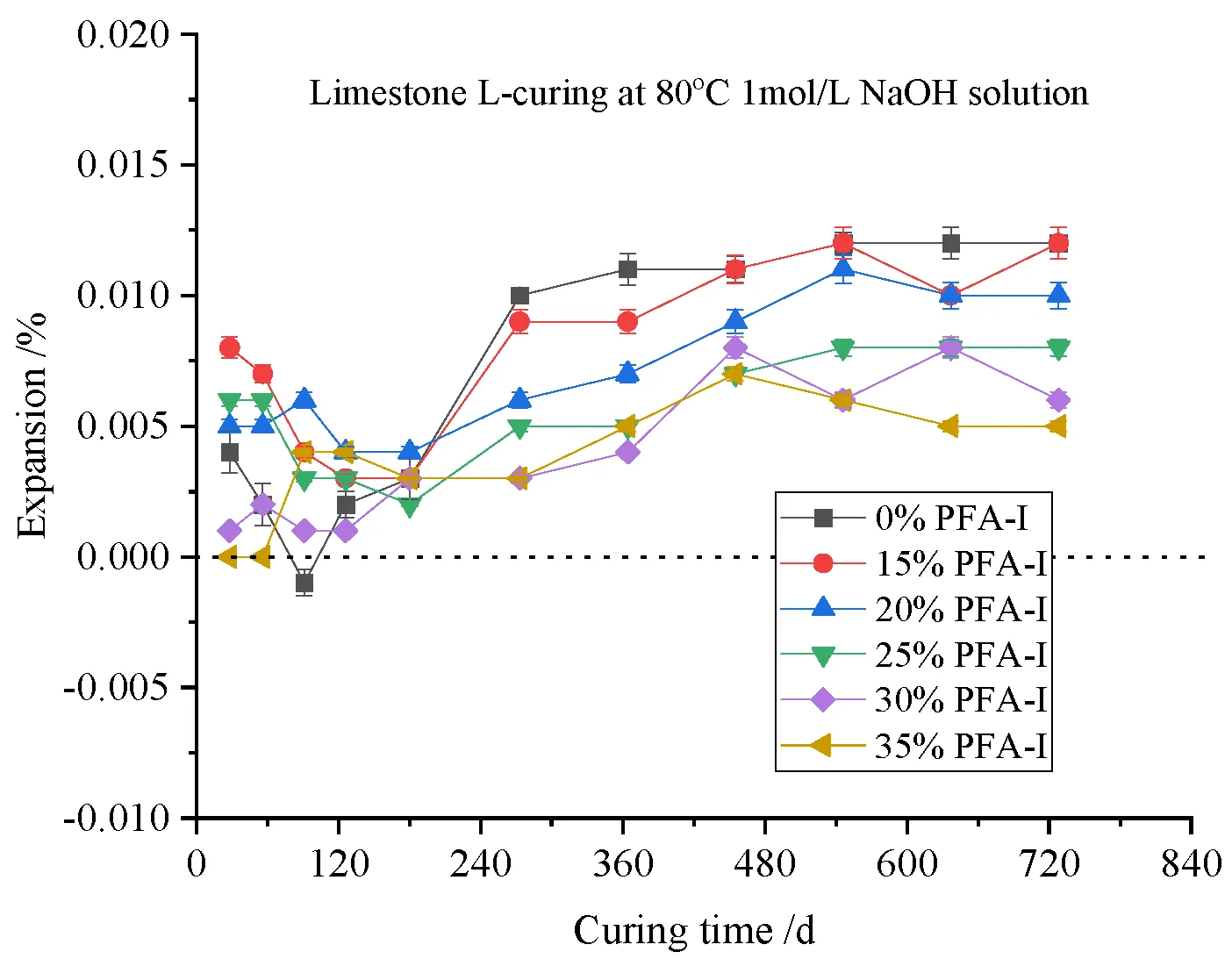
Effect of fly ash replacement on ACPT expansion of limestone aggregate during immersion in 1 mol/L NaOH at 80 °C.

Immersion in 1 mol/L NaOH at 80 °C caused faster and much larger expansion than standard CPT (Figure 8). With the increase in curing durations, the expansion of the concrete prisms also increased. The limestone mixtures showed only small changes and did not exceed 0.012%(Figure 9).

Fly ash reduced ACPT expansion, but the effective dosage increased with test age. At 182 d, 20% fly ash kept every tuff-bearing prism below 0.040%, with a maximum expansion of 0.036%. At 273 d, the minimum effective level increased to 30%, with a maximum expansion of 0.037%. At 364 d, no dosage up to 35% met the limit for all four tuffs. With 35% fly ash, the SA3, SA4, and SA34 prisms expanded by 0.057%, 0.054%, and 0.043%, respectively.

ACPT made the differences among fly ash levels easier to distinguish. It also kept the concrete prism size and coarse aggregate grading. However, more mixtures failed as the test continued. This shows that the 0.040% limit must be linked to a fixed ACPT age. The high temperature and continuous external alkali supply make ACPT a severe comparison test.

### 3.3. Comparison of Test Methods

All three methods showed that Grade I Class F fly ash reduced the ASR expansion of the four reactive tuffs. However, the minimum fly ash replacement depended on the specimen type, aggregate size, curing condition, temperature, alkali supply, and test age. The results from each method should therefore be interpreted according to its test conditions.

The AMBT uses mortar bars prepared with crushed aggregate. The specimens are immersed in 1 mol/L NaOH at 80 °C. This condition produces rapid expansion and is suitable for preliminary screening. At 28 d, 15% fly ash kept the expansion of all four tuff mixtures below 0.10%. The required replacement increased with exposure time. It reached 30% at 91 d. At 150 d, even 35% fly ash could not keep all specimens below 0.10%. The continuous external alkali supply made the test increasingly severe at later ages.

The standard CPT uses concrete prisms and retains the coarse aggregate grading and concrete proportions. It is therefore closer to the material scale of concrete used in practice. For this reason, CPT is commonly used as the main laboratory method for evaluating ASR mitigation measures [3,25]. In the present study, 15% fly ash kept the 728 d expansion of all four tuff concretes below 0.040%. This replacement was therefore effective under the standard CPT condition. The main limitation of the standard CPT is its long test period. The prisms are stored at 38 °C under high relative humidity. Long-term control of temperature, humidity, and specimen moisture can be technically demanding. The moisture condition of the specimens may also be affected by their position, sealing condition, and operation of the curing chamber. Solution immersion provides a more uniform moisture boundary around the specimens.

The ACPT used concrete prisms with the same dimensions and aggregate grading as those used in the standard CPT. The specimens were immersed in 1 mol/L NaOH at 80 °C. The solution provided continuous moisture and external alkalis. The elevated temperature accelerated silica dissolution and ASR product formation. The method therefore retained the concrete specimen scale while increasing the reaction rate. A similar principle is used in the MCPT, which accelerates ASR while retaining the main features of concrete specimens [28,29,30]. High temperature and high alkalinity may also increase the reaction of fly ash [26]. The ACPT separated the effects of different fly ash replacement levels within a shorter period than the standard CPT. At 182 d, 20% fly ash kept the expansion of all four tuff concretes below 0.040%. The maximum expansion at this replacement level was 0.036%. At 273 d, the minimum replacement increased to 30%, and the maximum expansion was 0.037%. These results show that the ACPT can distinguish the performance of different fly ash levels within 6–9 months. At 364 d, 35% fly ash did not keep all four mixtures below 0.040%. This result shows that the ACPT outcome depends strongly on test age. Longer exposure to high temperature and external alkalis increased the severity of the test. Therefore, the ACPT age and expansion criterion must be defined together. The 0.040% criterion cannot be applied to different ACPT ages without considering the increase in exposure severity. Measurements were conducted at 28, 56, 91, 126, 182, 273, 364, 455, 546, 637, and 728 days to cover the full ASR evolution process. The 28-day age complies with the standard AMBT evaluation requirement. The ages of 56–126 days capture early ASR acceleration, while 182 days and 273 days serve as critical evaluation stages for the accelerated ACPT. The long-term ages from 364 to 728 days correspond to the standard CPT protocol, allowing continuous monitoring of slow, sustained concrete expansion. This multi-age scheme ensures comprehensive comparison of ASR characteristics and fly ash mitigation effects across different test methods.

Table 3 summarizes the minimum fly ash replacement obtained from AMBT, standard CPT, and ACPT at different assessment ages. The required replacement increased with exposure time in both accelerated methods. In contrast, 15% fly ash satisfied the standard CPT criterion after 728 d. The AMBT results show that the usual 28 d assessment gave the same minimum replacement as the standard CPT. However, longer NaOH exposure increased the required replacement from 15% to more than 35%. A similar age effect was observed in ACPT. The minimum replacement increased from 20% at 182 d to 30% at 273 d. These results confirm that the assessment age must be specified when an accelerated method is used to evaluate fly ash mitigation.

The standard CPT remains the main reference because it uses concrete specimens and a recognized long-term curing condition. The ACPT provides results within a shorter period and offers a more uniform moisture and alkali boundary. It can be used to compare fly ash replacement levels before the standard CPT is completed. For engineering applications, the ACPT result should be considered together with the standard CPT result, the exposure condition, and the required service life.

### 3.4. Alkali–Silica Reaction of Tuff Aggregate in Concrete Prisms

Concrete prisms prepared with P·N 42.5 cement and SA3 tuff aggregate were examined after 2 years of exposure. The specimens were stored either in moist air at 38 °C or in 1 mol/L NaOH solution at 80 °C. The reaction morphology, surface deposits, and visibly reacted depth of the tuff aggregate were evaluated.

#### 3.4.1. Reaction of Tuff Aggregate Under Moist-Air Curing at 38 °C

**Figure 10:**
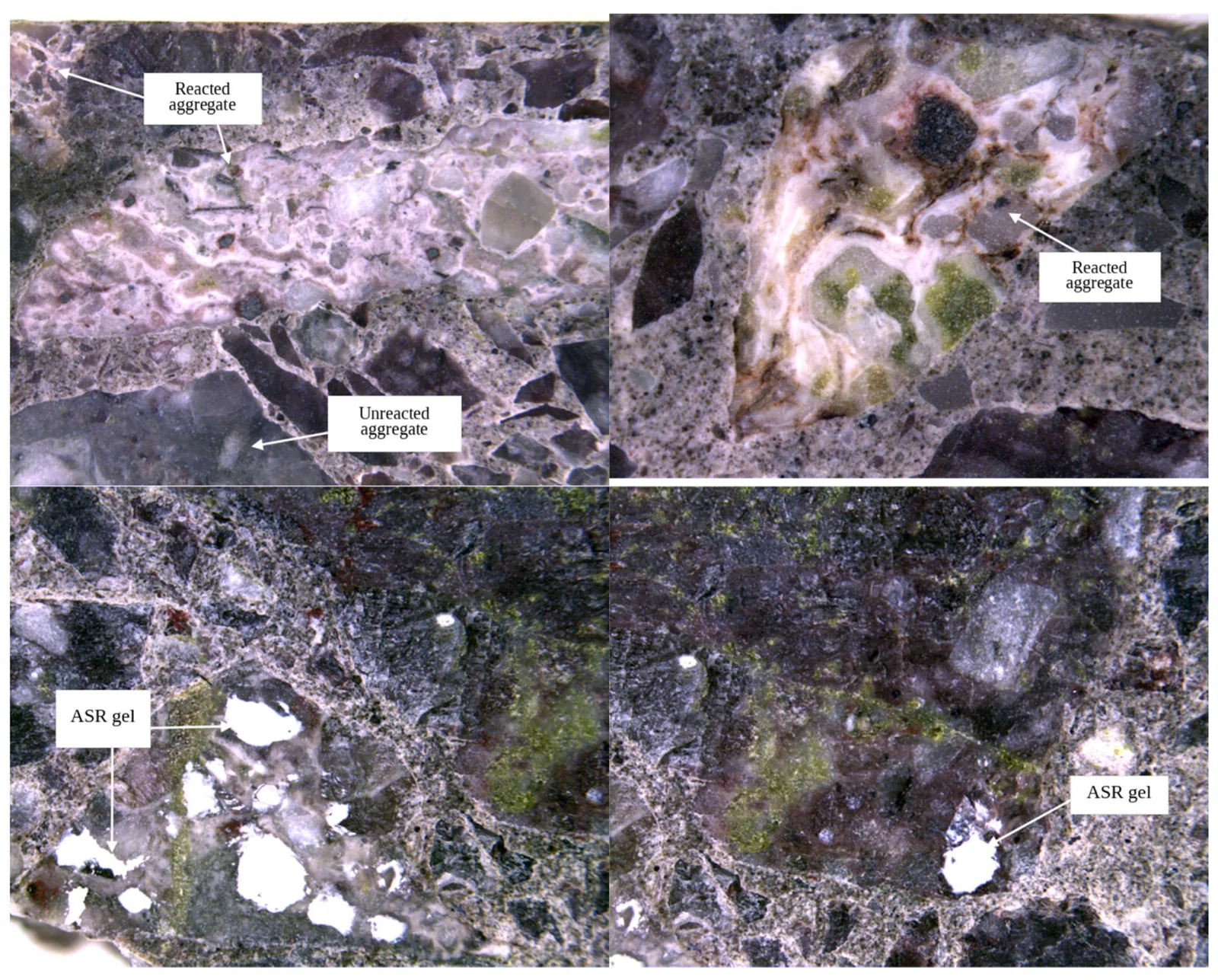
Reaction morphology of SA3 tuff aggregate in a concrete prism without fly ash after 2 years in moist air at 38 °C. Reacted and unreacted aggregate particles and ASR gel are marked.

Figure 10 shows the aggregate morphology in the SA3 concrete without fly ash after 2 years of curing in moist air at 38 °C. A small number of tuff particles showed clear reaction features. The local aggregate matrix became lighter, and white deposits were observed on some aggregate surfaces and in adjacent areas.

**Figure 11:**
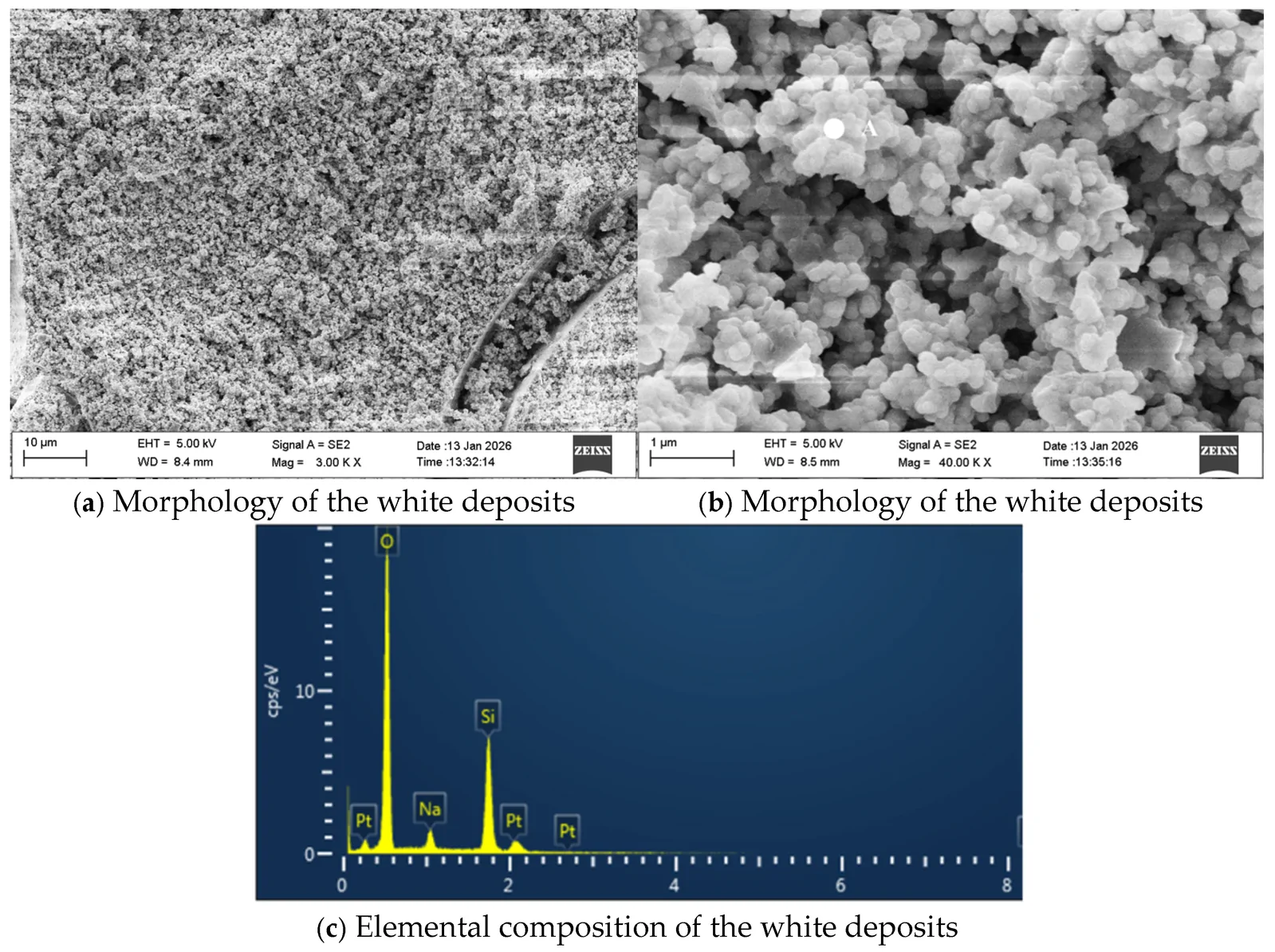
SEM morphology and elemental composition of white deposits in SA3 tuff aggregate in a concrete prism without fly ash after 2 years in moist air at 38 °C.

Figure 11 shows the SEM morphology and EDS spectrum of the white deposits. The deposits consisted of fine agglomerated particles and contained mainly Si, Na, and Ca. Based on their morphology and elemental composition, the deposits were identified as ASR gel.

#### 3.4.2. Reaction of Tuff Aggregate During Immersion in 1 mol/L NaOH at 80 °C

**Figure 12:**
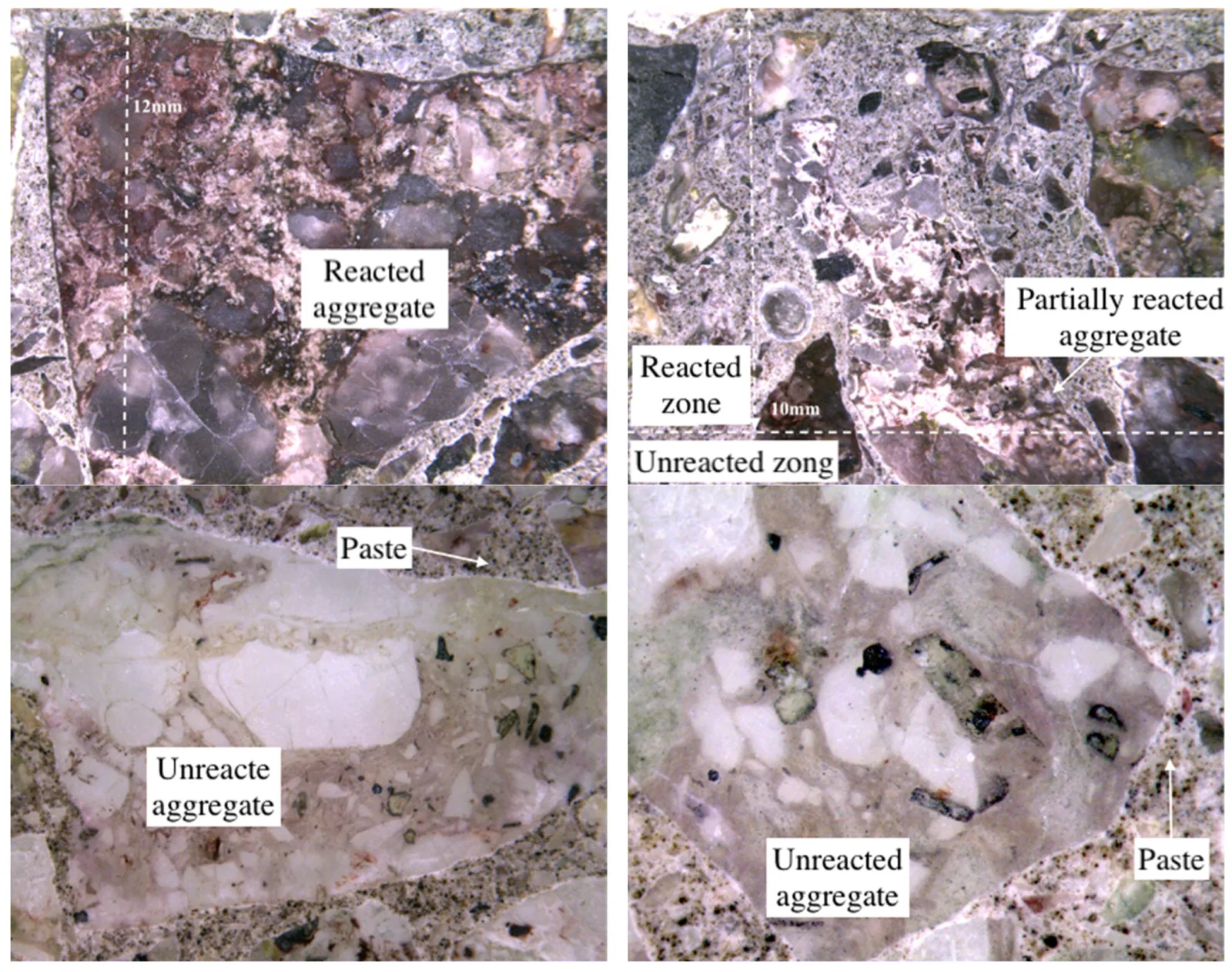
Reaction of SA3 tuff aggregate in a concrete prism with 0% fly ash after 2 years of immersion in 1 mol/L NaOH at 80 °C. Clearly reacted, partially reacted, and unreacted regions are marked.

Figure 12 shows the SA3 concrete without fly ash after 2 years of immersion in 1 mol/L NaOH at 80 °C. Within about 12 mm of the specimen surface, parts of the tuff matrix became white, and clearly reacted, partially reacted, and unreacted regions could be distinguished. No clear change was observed in tuff particles at depths greater than 12 mm.

**Figure 13:**
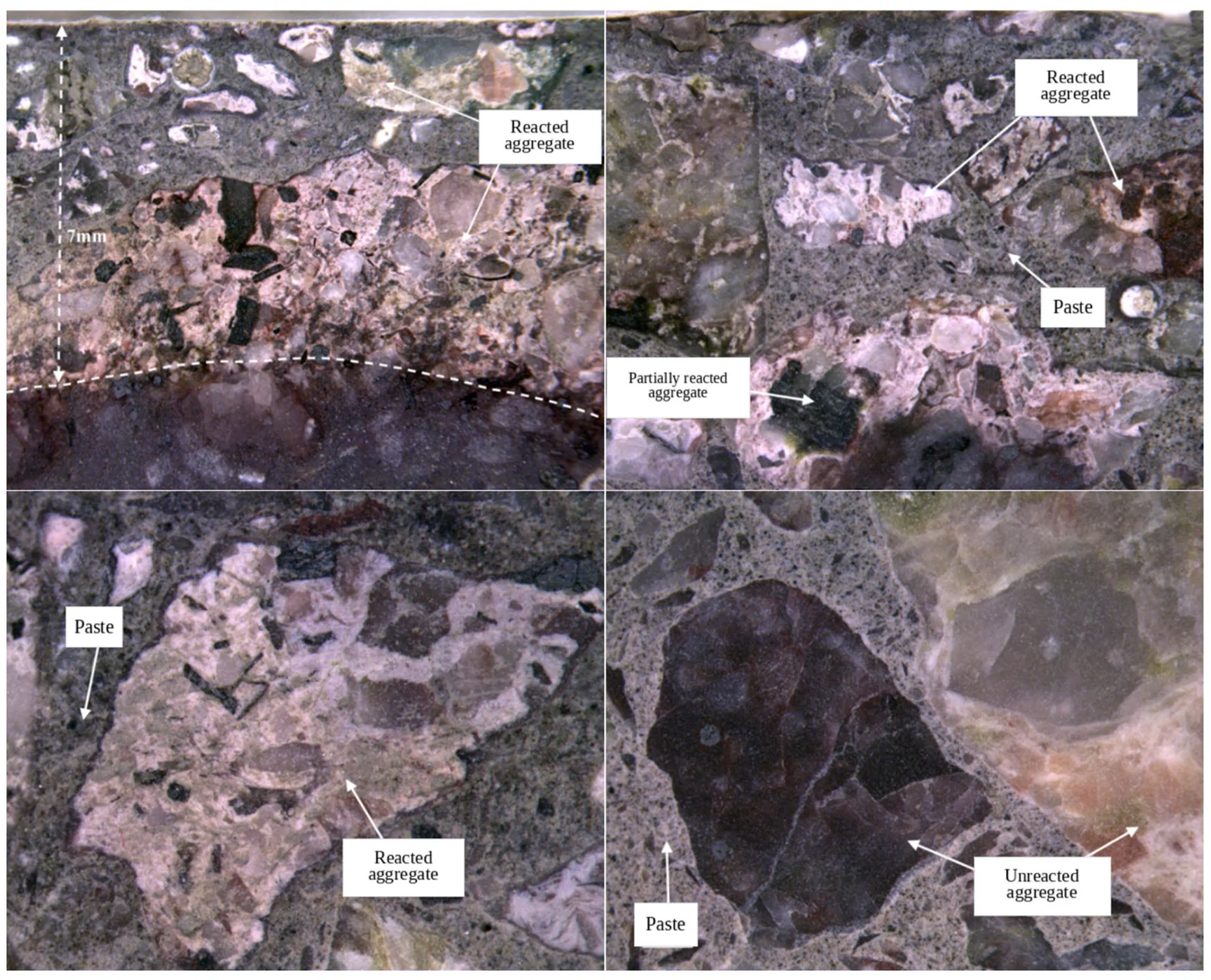
Reaction of SA3 tuff aggregate in a concrete prism with 15% fly ash after 2 years of immersion in 1 mol/L NaOH at 80 °C. Clearly reacted, partially reacted, and unreacted regions are marked.

With 15% fly ash, the visibly reacted tuff particles were mainly located within about 7 mm of the specimen surface, as shown in Figure 13. Beyond this depth, the aggregate generally retained its original colour and structure.

**Figure 14:**
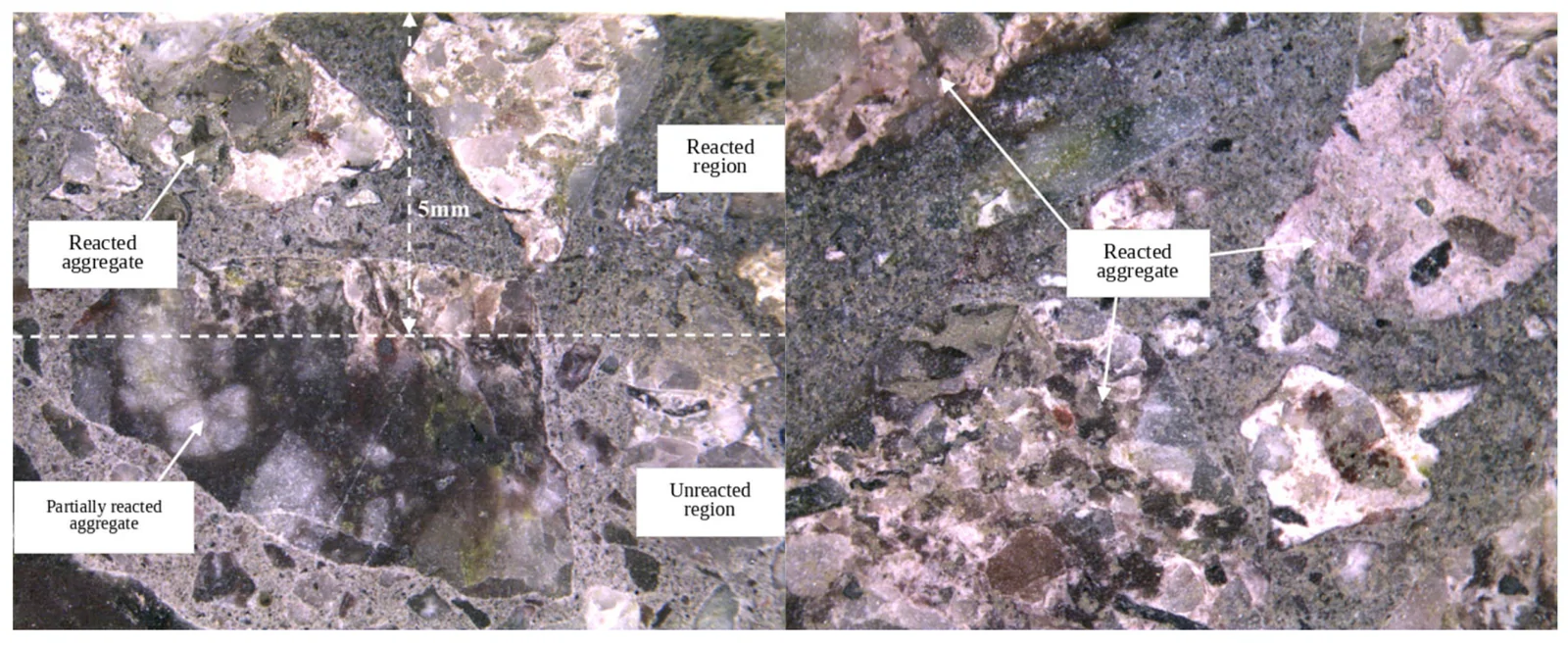

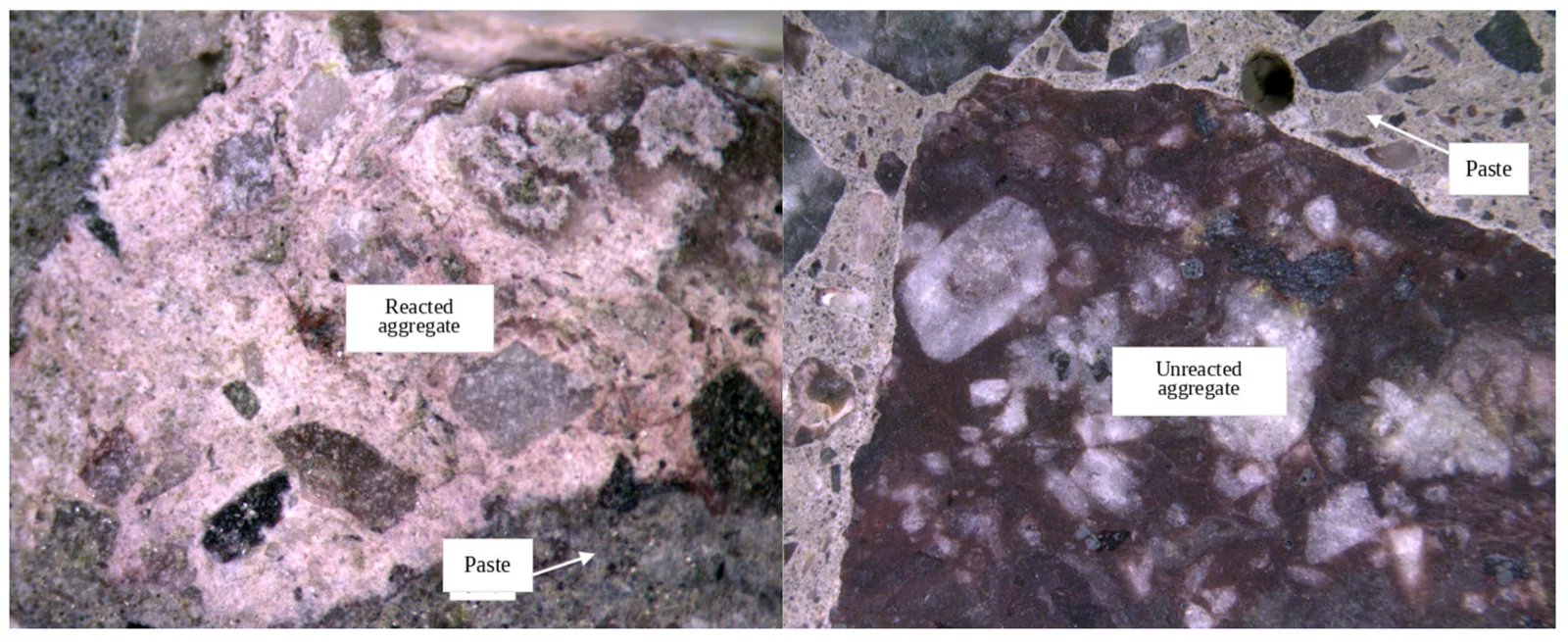
Reaction of SA3 tuff aggregate in a concrete prism with 35% fly ash after 2 years of immersion in 1 mol/L NaOH at 80 °C. Clearly reacted, partially reacted, and unreacted regions are marked.

At a fly ash replacement of 35%, the visibly reacted region decreased further to about 5 mm from the specimen surface, as shown in Figure 14. No clear reaction was observed in tuff particles beyond this depth.

As the fly ash replacement increased from 0% to 15% and 35%, the visibly reacted depth decreased from about 12 mm to 7 mm and 5 mm, respectively.

#### 3.4.3. Relationship Between Tuff Reaction and Concrete Expansion

The reaction morphology of the tuff aggregate followed the same general trend as the concrete prism expansion. After 2 years in moist air at 38 °C, local tuff reaction and ASR gel were observed in the SA3 concrete without fly ash. Its 728 d expansion was 0.053%. With 15% fly ash, the expansion decreased to 0.018%.

After 2 years in 1 mol/L NaOH at 80 °C, the visibly reacted depth decreased from about 12 mm without fly ash to 7 mm and 5 mm at fly ash replacements of 15% and 35%, respectively. The ACPT expansion also decreased as the fly ash replacement increased. These results indicate that fly ash limited the inward penetration of external alkalis, restricted the development of the reacted tuff region, and reduced ASR expansion.

The ASR depth is defined as the radial thickness of the reaction rim formed by alkali penetration and silica dissolution from the aggregate–paste interface into the reactive tuff grain. Alkali ions diffuse inward through pores and microcracks, dissolving amorphous volcanic glass and precipitating ASR gel at the reaction front. New microcracks induced by gel swelling further accelerate alkali ingress, leading to non-linear growth of reaction depth with exposure time [4,21]. Supplementary cementitious materials such as fly ash retard the advance of reaction front by lowering pore alkalinity and refining pore structure [11,22].

### 3.5. Limitations and Future Prospects

This study has several inherent limitations that should be acknowledged. First, all ASR evaluations were performed under laboratory-controlled conditions. The ACPT adopts a high-temperature and high-alkali accelerated environment, which cannot fully reproduce the complex in situ service conditions of concrete, such as variable temperature–humidity cycles, leaching of alkalis, and combined chemical attacks. Second, only four types of tuff aggregates from Southern Zhejiang and one Grade I Class F fly ash were tested. The findings may not be directly extrapolated to tuff aggregates with different mineralogical compositions or fly ash of varying reactivity and fineness. Third, the measured reaction depth was obtained from post-mortem microscopic observation of specimens at specific ages. The temporal evolution of the reaction front and chemo-mechanical coupling cracking were not quantitatively modelled in the present work.

Future research can be extended in several directions. Long-term field exposure tests are recommended to calibrate the accelerated test results from ACPT and establish reliable conversion factors between laboratory acceleration and field performance. Further work could explore the synergistic inhibition effect of fly ash combined with other supplementary cementitious materials, such as silica fume and metakaolin, on tuff ASR. In addition, multi-scale chemo-mechanical numerical models can be developed to simulate the transport of alkali ions, ASR gel formation, reaction front propagation, and crack initiation in tuff concrete. Moreover, the ASR behaviour under coupled loading and environmental actions (e.g., freeze–thaw, external sulfate attack) should be investigated to support the engineering application of reactive tuff aggregates in hydraulic and civil infrastructure.

## 4. Conclusions

This study compared the effect of fly ash on ASR expansion in four reactive tuffs. Three methods were used: AMBT, standard CPT, and ACPT in alkaline solution. The main conclusions are:

(1) Both AMBT and standard CPT results demonstrated that 15% replacement of Grade I Class F fly ash can effectively suppress the ASR expansion of the four reactive tuff aggregates. According to the AMBT results, the 28-day expansion of mortar specimens was significantly reduced from 0.212 to 0.376% (without fly ash) to 0.036–0.050% (with 15% fly ash), with all values well below the 0.10% critical threshold for deleterious ASR expansion. Consistent with the AMBT findings, the standard CPT results showed that the 728-day concrete expansion decreased from 0.037 to 0.064% to 0.018–0.029%, satisfying the strict 0.040% limit for durable concrete structures. Therefore, 15% fly ash replacement is verified as an effective dosage for mitigating tuff-related ASR expansion under conventional laboratory test conditions.
(2) The newly adopted ACPT method retained the original concrete prism geometry and full-size coarse aggregate used in standard CPT. The accelerated exposure condition of 1 mol/L NaOH solution at 80 °C provided a uniform alkali and moisture environment, which greatly accelerated the ASR process and enabled rapid differentiation of the mitigation efficiency of different fly ash dosages. Based on the ACPT results, the minimum fly ash replacement required to control the expansion of all four tuff concretes below the 0.040% limit was 20% at 182 days and 30% at 273 days. Compared with the conventional long-term CPT and short-term AMBT, the prolonged accelerated ACPT condition imposes a stricter evaluation criterion for ASR resistance. For engineering structures with high safety and long-term durability requirements, the 273-day ACPT result (30% fly ash replacement) is recommended to provide a sufficient safety margin for suppressing potential long-term ASR deterioration.
(3) Microstructural observations showed that fly ash reduced ASR expansion by restricting the inward transport of external alkalis through the concrete to the tuff aggregate, thereby limiting the reacted region. As the fly ash replacement increased from 0% to 15% and 35%, the visibly reacted depth of the tuff aggregate decreased from approximately 12 mm to 7 mm and 5 mm, respectively. The reduced reaction depth verifies that fly ash can densify the concrete microstructure and limit alkali ingress, which fundamentally alleviates the expansion and deterioration of reactive tuff concrete.

## Figures and Tables

**Table 1:** Chemical compositions of the reactive tuff aggregates.

No	Aggregate ID	Chemical Composition/wt.%									
		SiO_2_	Fe_2_O_3_	Al_2_O_3_	CaO	MgO	Na_2_O	K_2_O	SO_3_	LOI	SUM
1	SA3	70.07	2.89	14.32	2.20	0.61	2.09	2.61	0.08	2.70	97.56
2	SA4	71.50	2.87	13.37	2.18	0.72	2.56	2.78	0.10	1.14	97.22
3	SA34	69.78	2.88	15.50	3.21	0.42	2.32	2.58	0.06	1.68	98.43
4	SA6-R4	71.09	3.16	13.50	3.41	0.74	2.46	2.22	0.08	0.93	97.59

**Table 2:** Chemical compositions of cement and Grade I Class F fly ash.

No	Samples	Chemical Composition/wt.%									
		SiO_2_	Fe_2_O_3_	Al_2_O_3_	CaO	MgO	Na_2_O	K_2_O	SO_3_	LOI	SUM
1	Cement	21.39	4.24	4.46	62.16	2.14	0.14	0.37	2.62	0.54	98.06
2	Fly ash	48.84	7.69	29.33	6.98	1.76	0.85	0.88	0.91	0.56	97.80

**Table 3:** Minimum fly ash replacement determined under different test conditions and assessment ages.

Test Method and Curing Condition	Test Age	Expansion Criterion	Minimum Fly Ash Replacement for All Tuffs	Maximum Expansion at the Stated Replacement
AMBT, 80 °C and 1 mol/L NaOH solution	28 d	0.10%	15%	0.050%
	56 d	0.10%	15%	0.090%
	91 d	0.10%	30%	0.083%
	120 d	0.10%	35%	0.090%
	150 d	0.10%	>35%	0.129% at 35% fly ash
Standard CPT, 38 °C and approximately 99% RH	728 d	0.040%	15%	0.029%
ACPT, 80 °C and 1 mol/L NaOH solution	182 d	0.040% *	20%	0.036%
	273 d	0.040% *	30%	0.037%
	364 d	0.040% *	>35%	0.057% at 35% fly ash

* Note: This is the minimum dosage adopted in the experiments.

## Data Availability

The original contributions presented in this study are included in the article. Further inquiries can be directed to the corresponding authors.

## Abbreviations

ACPT, Accelerated Concrete Prism Test; AMBT, Accelerated Mortar Bar Test; ANOVA, Analysis of Variance; ASR, Alkali–Silica Reaction; CPT, Concrete Prism Test; EDS, Energy-Dispersive X-ray Spectroscopy; FESEM, Field Emission Scanning Electron Microscope; SD, Standard Deviation; XRD, X-ray Diffraction; XRF, X-ray Fluorescence.
